# The usefulness of the Spanish version of the STOP-Bang questionnaire for screening for moderate or severe sleep apnea syndrome in primary care

**DOI:** 10.3389/fpubh.2022.975114

**Published:** 2022-09-09

**Authors:** Rafaela Muñoz-Gómez, Esther Navarrete-Martínez, Jesús Serrano-Merino, Fátima Silva-Gil, Ana Roldán-Villalobos, Enrique Martín-Rioboó, Javier Ruiz-Moruno, Esperanza Romero-Rodríguez, Jesus Gonzalez-Lama, Manuel Vaquero-Abellán, Luis Angel Perula-de-Torres

**Affiliations:** ^1^Centro de Salud Sector Sur, Distrito Sanitario Córdoba-Guadalquivir, Distrito Sanitario Córdoba Guadalquivir, Córdoba, Spain; ^2^Instituto Maimónides de Investigación Biomédica de Córdoba, Hospital Reina Sofía, Universidad de Córdoba, Córdoba, Spain; ^3^Centro de Salud de Marchena, Área de Gestión Sanitaria Osuna-Sureste Sevilla, Sevilla, Spain; ^4^Centro de Salud Sector Sur, Distrito Sanitario Córdoba-Guadalquivir, Córdoba, Spain; ^5^Centro de Salud Pedro Abad, Distrito Sanitario Córdoba-Guadalquivir, Córdoba, Spain; ^6^Centro de Salud Castilla del Pino, Distrito Sanitario Córdoba-Guadalquivir, Córdoba, Spain; ^7^Centro de Salud Poniente, Distrito Sanitario Córdoba-Guadalquivir, Córdoba, Spain; ^8^Centro de Salud Aeropuerto, Distrito Sanitario Córdoba-Guadalquivir, Córdoba, Spain; ^9^Unidad docente multiprofesional de atención familiar y comunitaria, Distrito Sanitario Córdoba-Guadalquivir, Córdoba, Spain; ^10^Centro de Salud de Cabra, Área de Gestión Sanitaria Sur de Córdoba, Córdoba, Spain; ^11^Facultad de Medicina y Enfermería, Universidad de Córdoba, Cordoba, Spain

**Keywords:** primary care (PC), obstructive sleep apnea syndrome (OSA), STOP-Bang questionnaire (SBQ), home respiratory polygraphy (HRP), Berlín questionnaire (BQ)

## Abstract

**Rationale:**

Sleep apnea-hypopnea syndrome (OSA) is a highly prevalent disease and has been related to cardiovascular diseases and occupational and traffic accidents. Currently, it is estimated that there is a significant underdiagnosis of OSA, mainly due to the difficulty accessing the tests for that purpose.

**Objective:**

To determine the usefulness of the Spanish version of the STOP-Bang questionnaire (SBQ) for screening for moderate or severe OSA in the adult population attending primary care.

**Methods:**

A descriptive observational multicenter study was conducted. Through an opportunistic search, (patients over 18 years old), were recruited in seven primary care centers. The SBQ was applied to them and home respiratory polygraphy (HRP) was subsequently performed to confirm the diagnosis of OSA. The criterion validity of the SBQ was analyzed, comparing the score obtained by the SBQ with the apnea-hypopnea index (AHI) obtained by RP, establishing the diagnosis of OSA for an AHI>5. The reliability of the questionnaire was evaluated.

**Results:**

A total of 255 subjects, 54.1% men, with a mean age of 54.76 ± 10 years, were recruited in the study. The results showed that 61.57% (95% Confidence Interval: 55.57–67.57) of the subjects presented OSA, presenting 22.75% (17.57–57.92) a mild OSA (530) (11.54–20.62). The Kuder and Richardson coefficient was 0.623 (0.335–0.788) and Cohen's Kappa coefficient was 0.871 (0.520–1.00; *p* < 0.001). For moderate/severe OSA screening (AHI>15) the SBQ obtained an ROC curve of 0.769 (0.704–0.833) that with an optimal cutoff of 3, achieved a sensitivity of 84.85% (77.28–92.42) and a specificity of 55.10% (44.74–65.46).

**Conclusions:**

The SBQ is very effective for detecting moderate/severe OSA. Its psychometric properties are similar to those obtained in studies on other populations. Because of its ease of use, the SBQ is a very useful tool for primary health care professionals.

## Introduction

Sleep apnea-hypopnea syndrome (OSA) is a chronic disease that causes upper airway (UA) collapse resulting in multiple episodes of complete (apnea) or partial (hypopnea) obstruction, causing poor sleep quality and intermittent hypoxemia with vascular impact (1).

OSA prevalence in the general population is between 6 and 10% (2), being higher with increasing age. In Spain, between 1,200,000 and 2,150,000 people suffer from OSA, and of them, between 24 and 26% (3) present a very severe picture, being, in addition, OSA a condition related to cardiovascular diseases and occupational and traffic accidents (4).

However, it is currently estimated that only 5–9% of people with OSA have been diagnosed, with lack of accessibility to a diagnosis being the main cause attributed (5, 6).

Conventional polysomnography (PSG) is the gold-standard method for diagnosing OSA. PSG consists of continuous recording during the sleep period of neurophysiological (electroencephalogram, electrooculogram, and mental electromyogram), respiratory (peripheral saturation of O_2_ and oronasal airflow by nasal cannula and/or thermistor), and other parameters (snoring, thoracoabdominal musculature movements, electrocardiogram, leg movement, and position); however, the realization of PSG requires a costly hospital infrastructure that not all health systems can cover (7). An alternative to PSG is home respiratory polygraphy (HRP). It consists of recording respiratory variables (peripheral O_2_ saturation and airflow) and other variables (snoring, thoracoabdominal muscle movements, and position) in the patient's home *via* portable equipment. This method of diagnosis of OSA has demonstrated good psychometric properties in different health care settings, especially in patients with suspected moderate or severe OSA (7–10).

The search to improve accessibility to the diagnosis of OSA highlights the need to implement new formulas for early detection, the approach, and management of this condition, mainly from the primary care setting (2, 11, 12). In this sense, the STOP-Bang questionnaire (13) has proved its validity and reliability in various healthcare settings (14). However, at present, there are few studies using the Spanish version that support and have demonstrated with the necessary evidence its validity and reliability in primary care patients (15); therefore, we understand pertinent to perform more studies that demonstrate the clinical utility of this questionnaire.

## Methodology

### Design

Descriptive observational study of validation of the Stop-Bang questionnaire as a valid and reliable measurement tool compared to HRP (gold standard).

The study population consisted of people recruited from seven primary care centers of the Cordoba-Guadalquivir Health District (Cordoba, Spain); 5 were urban and 2 were rural.

The inclusion criteria were: People aged 18 years or above, of both sexes, who attended their health center for any reason and signed the informed consent. The following were considered exclusion criteria: Patients with a previous diagnosis of OSA, those who, because of disease, cognitive status, or educational level, were unable to answer the STOP-Bang questionnaire, and patients on hypnotic treatment or with chronic alcoholism.

Recruitment was conducted by consecutive sampling, opportunistically, offering participation to those individuals who met the selection criteria until the sample size was completed. Applying results from previous studies (15) and using the Epidat 3.1 statistical package, for a sensitivity of 84%, a non-sick/sick ratio of 0.160, an absolute accuracy of 6.2%, and a confidence level of 95%, the sample size required for conducting our study was 157 people: 135 sick and 22 non-sick.

The study variables were: Age, gender, body mass index (BMI = weight in kg/height in square meters) (16), apnea-hypopnea index (AHI), defined as the number of apneas plus hypopneas per 1 h of polygraphy or polysomnographic (3) study, and the 8 items constituting the STOP-Bang questionnaire (13).

The recruited people were referred in <1 week to a researcher expert in sleep-related breathing disorders. Once they attended the appointment, the researcher detailed the study characteristics and development, answered the possible doubts, and after the signing of the consent to participation, the data were collected, starting with measuring the weight and height using a scale/stadiometer “Seca 711 class III”. The precision of the weight measurement was 0.10 kg and of the height was 0.5 cm. Additionally, the neck circumference was measured, with a tape measure on its flat side, without exerting any pressure on the skin, excluding the hair and surrounding the neck, passing through the area of Adam's apple. A flexible fiberglass tape measure of 3 cm wide and 120 cm long, OEM brand, was used, and the measurement accuracy was 0.2 cm. All measurements were performed in triplicate, considering the arithmetic mean of the measurements as a reference. Then, the people completed the STOP-Bang questionnaire. Finally, a polygraph was given to each subject, and they were trained to use it, performing an on-site simulation of the placement of all electrodes, ensuring that the patients had assimilated all the information. The doubts were resolved, telephone contact was provided, and they were summoned again to deliver the polygraph the next day. The polygraph used was SCREENG&GO-Sibelmed, with 6 channels (air flow, thoracoabdominal movements, snoring, body position, pulse, and oxygen saturation). The time of registration for each study was 6 h, with a study considered valid when it recorded at least 3 h of registration (3). A total of 16 out of the 255 HRP performed were not recorded correctly, so they were repeated the following day, obtaining valid values on this second occasion. Polygraph studies were automatically analyzed by the polygraph software “Bitmelad.” After 1–3 days, they were manually analyzed by the researcher expert in sleep-related breathing disorders following the Spanish Society of Pulmonology and Thoracic Surgery (SEPAR) criteria (3) and without being aware of the result of the STOP-Bang questionnaire completed by each subject. The diagnosis of OSA was established through the results of the home HRP, considering the existence of OSA for AHI > 5 (3). OSA was classified (3) as: Mild for 5 < AHI ≤ 15, moderate for 15 < AHI ≤ 30, and severe for AHI > 30.

A collaborating researcher, different from the one who previously collected the data, randomly selected a subsample of 31 people from the entire study to assess the reproducibility or reliability of the STOP-Bang questionnaire in terms of the interobserver agreement. She contacted them by phone within 3 months of the first data collection, and they populated the STOP-Bang questionnaire again.

### The STOP-Bang questionnaire

The STOP-Bang questionnaire (13) is an easy-to-complete OSA screening tool. The acronym of this questionnaire stands for: “S” snore, “T” tired, “O” observed apneas, “P” pressure, “B” BMI (body mass index >35 kg/m^2^), “A” age (age > 50 years), “N” neck (neck circumference >43 cm in men or >41 cm in women), and “G” gender (male gender). Each of these 8 items is collected as a dichotomous question (YES/NO), adding 1 point for each question answered as “yes.” A score of 0 to 2 is considered a low risk of OSA, a score of 3 to 4 is a moderate risk of OSA, and a score of 5 or higher is at high risk for OSA.

### Statistical analysis

The statistical package SPSS v.19 was used. The descriptive analysis was performed for quantitative variables, and the absolute and relative frequencies for the different groups were tabulated for qualitative variables, expressing the most significant statistics with their confidence intervals of 95% of safety (95% CI). A bivariate analysis was performed for the OSA gender and OSA grade variables using the Pearson Chi-square test, a *p*-value below 0.05 was considered significant. The internal consistency was determined through the Kuder and Richardson index (17), interpreting the results according to Oviedo and Campo (18). The concordance between observers was evaluated by using Cohen's Kappa coefficient (19), comparing the results of the STOP-Bang questionnaire that was administered twice, by two different researchers, a random subsample of 31 individuals, interpreting the degree of agreement according to the Landis and Koch scale (20). The AHI obtained by polygraphy was compared with the sum of the STOP-Bang questionnaire scores, calculating the area under the ROC curve (AUC) and determining the optimal cutoff points, performing the analysis by gender. The values of sensitivity, specificity, positive predictive value (PPV), negative predictive value (NPV), positive likelihood ratio (LR +), and negative likelihood ratio (LR –) with their corresponding 95% CIs were calculated.

### Ethical considerations

The study has obtained the approval of the Research Ethics Committee of Cordoba (Act No. 279, ref. 3915) and the authorization of the officials responsible for the Cordoba and Guadalquivir Health District. The study complies with the principles established in the Declaration of Helsinki, the Convention of the Council of Europe on human rights and biomedicine, and the requirements established in Spanish legislation. The study also complied with the good clinical practice standards (art. 34 RD 223/2004; Community Directive 2001/20/EC), the Law on Personal Data Protection and Guarantee of Digital Rights (Organic Law 3/2018, of 5 December), the Law on Patient Autonomy 41/2002 and the Law on Biomedical Research 14/2007.

## Results

The final number of patients recruited was 255 people, of whom 138 (54.1%) were men and 117 (45.9%) were women. The mean ± standard deviation (SD) of age was 54.76 ± 10 years (95% CI: 53.53–55.59), BMI 31.17 ± 6.58 kg/m^2^ (95% CI: 30.36–31.98), and neck circumference 38.33 ± 5.78 cm (95% CI: 37.62–39.05; 95% CI), with significant differences in neck circumference and BMI by gender (Table 1).

**Table 1:** Anthropometric variables.

	**Men**			**Women**			***p*-value**
	***n** =* **138 (54.1%)**			***n** =* **117 (45.9%)**			
	**Mean (SD)**	**CI (95%)**	**Median**	**Mean (SD)**	**CI (95%)**	**Median**	
Age	55.57 (10.10)	53.87–57.27	55.5	53.80 (9.82)	52.00–55.60	54	0.165(*)
BMI	32.39 (6.49)	31.29–33.48	31.07	29.73 (6.41)	28.56–30.91	28.76	<0.001(**)
Neck circumference	41.51 (5.30)	40.62–42.41	41	34.58 (3.69)	33.90–35.26	34	<0.001(***)

Age, Years; BMI, Kg/m^2^; Neck circumference, cm; SD, Standard deviation; CI (95%), 95% confidence interval. (^*^), Mann-Whitney U = 14161.50. (^**^), Mann-Whitney BMI U = 5967.50. (^***^), T-Student = 2.302 (assuming equal variances [Levene > 0.05]).

The internal consistency, measured with the Kuder and Richardson coefficient, was 0.623 (95% CI: 0.541–0.665). The interobserver agreement, measured with Cohen's Kappa coefficient, was 0.936 (95% CI: 0.813–1.00; *p* < 0.001).

**Figure 1:**
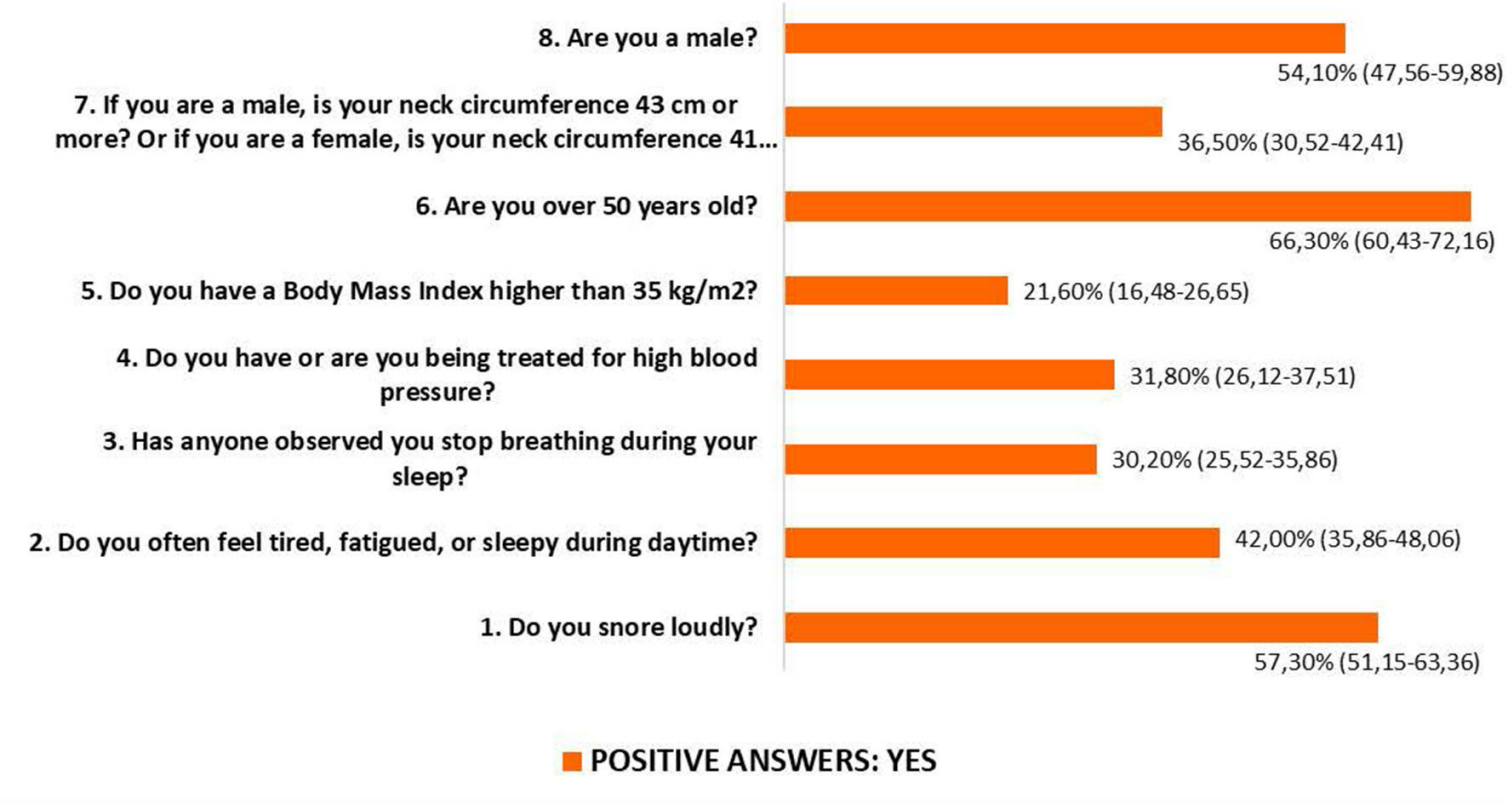
STOP-Bang results. Population parameters with a 95% confidence level. Item 1: YES 44.4% (95% CI: 35.30–53.58) of women and 68.1% (95% CI: 60.24–75.98) of men (*p* < 0.021). Item 2: YES 41% (95% CI: 31.98–50.07) of women and 42.8% (95% CI: 34.39–51.11) of men (*p* = 0.010). Item 3: YES 14.5% (95% CI: 8.04–21.01) of women and 43.5% (95% CI: 35.10–51.85) of men (*p* < 0.001). Item 4: YES 25.6% (95% CI: 17.61–33.67) of women and 37% (95% CI: 28.80–45.11) of men (*p* < 0.001). Item 5: YES 15.4% (95% CI: 8.74–22.01) of women and 26.8% (95% CI: 19.27–34.29) of men (*p* < 0.001). Item 6: YES 65.8% (95% CI: 57.08–74.53) of women and 66.7% (95% CI: 58.70–74.63) of men (*p* < 0.001). Item 7: YES 6.8% (95% CI: 2.19–11.47) of women and 61.6% (95% CI: 53.37–69.81) of men (*p* < 0.001). Total sample *n* = 255 (117[45.9%] women and 138 [54.1%] men), Pearson's Chi-square contrast statistic.

Significant gender differences existed in all responses to the STOP-Bang questionnaire (Figure 1).

**Figure 2:**
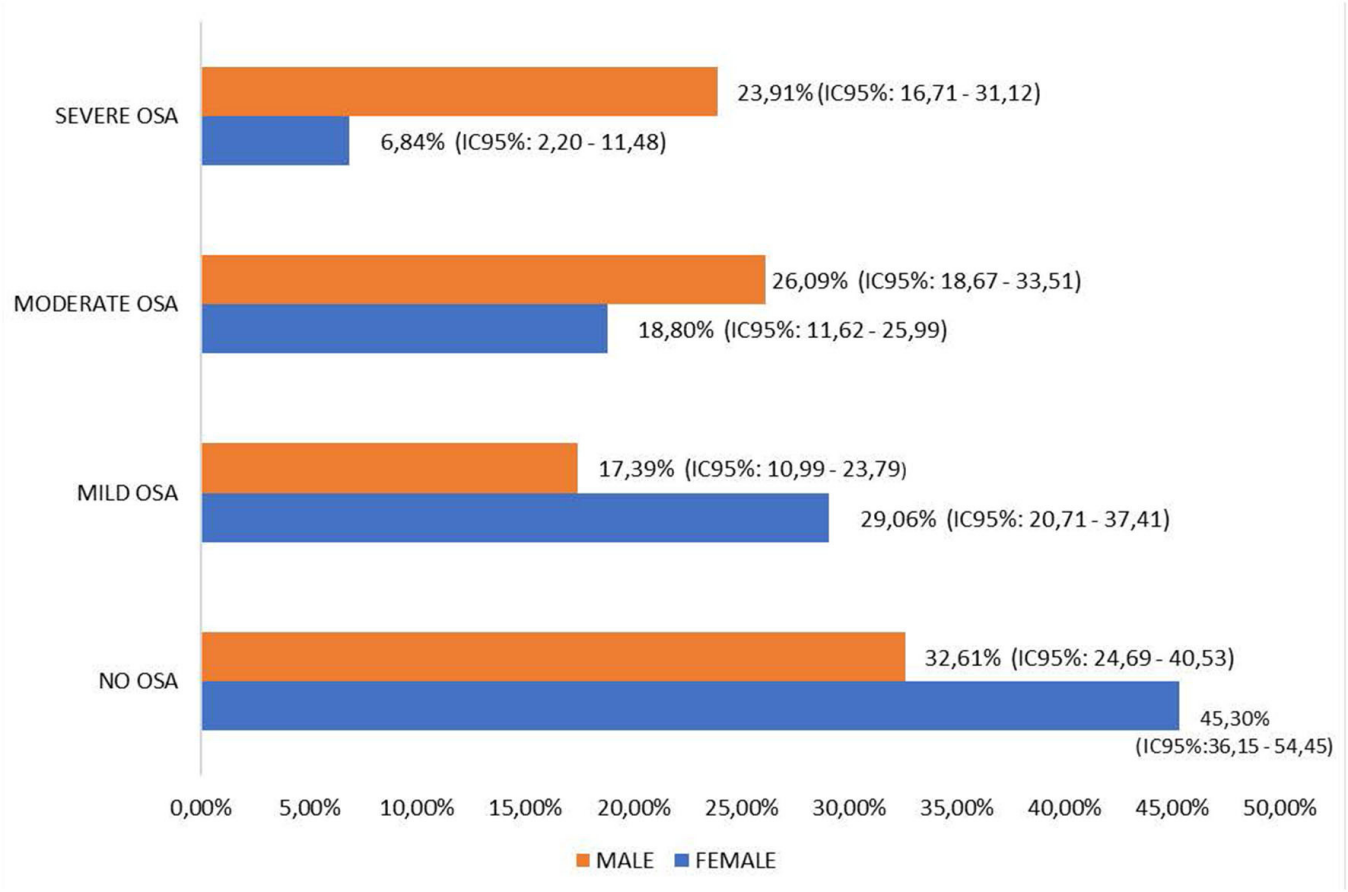
Distribution of OSA by gender. Population parameters with a 95% confidence level. NO OSA: Pearson's Chi square = 4.310, *p* = 0.038. MILD OSA: Pearson's Chi square = 4.906, *p* = 0.027. MODERATE OSA: Pearson's Chi square =1.191, *p* = 0.167. SEVERE OSA: Pearson's Chi square = 13.682, *p* < 0.001.

**Figure 3:**
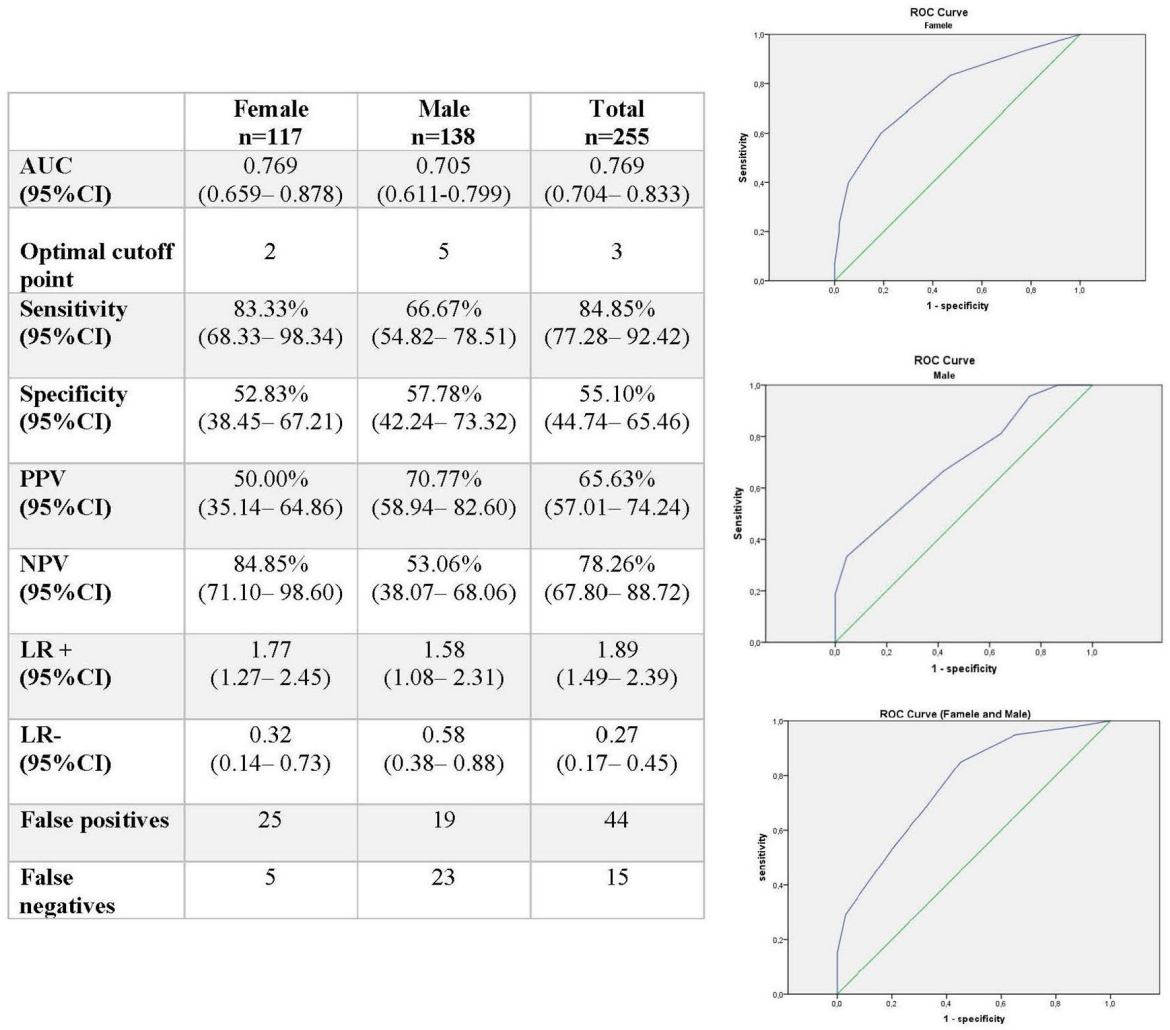
Criterion validity of the STOP-Bang questionnaire for the screening for moderate/severe apneas (Apnea-Hypopnea Index [AHI] >15). AUC, area under the curve; 95% CI, 95% confidence interval; LR, likelihood ratio; NPV, negative predictive value; PPV, positive predictive value.

A total of 38.43% (95% CI: 32.42–44.44) presented no OSA, with significant differences by gender (*p* = 0.038). Mild OSA was found in 22.75% (95% CI: 17.57–57.92) of people, 22.7% (95% CI: 17.57–57.92) of people presented moderate OSA, and 16.08% (95% CI: 11.54–20.62) presented severe OSA, with significant gender differences in mild OSA (*p* = 0.038) and severe OSA (*p* < 0.001) (Figure 2). The AUC for detecting AHI > 15 was 0.769 (95% CI: 0.704–0.833), with an optimum cutoff point of 3, providing a sensitivity of 84.85% (95% CI: 77.28–92.42) and a specificity of 55.10% (95% CI: 44.74–65.46) (Figure 3).

No significant differences were found by gender in the ability to diagnose true positives and true negatives (Chi-square = 2.18; *p* = 0.10).

## Discussion

In our study, for scores higher than or equal to 3, the STOP-Bang questionnaire showed a 77% diagnostic power for moderate/severe sleep apneas (AHI > 15), with an ability to classify true positives of 85% and true negatives of 55%. For the detection of an AHI > 5, the STOP-Bang sensitivity was 73.25% and specificity was 55.10%, with a good classification ability of 69.4%, results that should be considered with caution because respiratory polygraphy, which is recommended only for moderate to severe apneas (AHI > 15), was the gold standard used for comparisons.

Although Oviedo and Campo (18) indicate an internal consistency above 0.70 as optimal, we consider that a Kuder and Richarson coefficient (17) of 0.623 indicates an acceptable internal consistency considering that we are evaluating the coherence of the questionnaire components to determine the extent to which this tool reflects the theory of the phenomenon to be measured and that this tool has been developed by experts and validated for other populations. According to Landis and Koch (20), the interobserver agreement range obtained was “almost perfect,” confirming the reproducibility of the STOP-Bang questionnaire.

The results of our study were similar to those reported by others performed in Spanish-speaking primary care populations. Cruces-Artero et al. (15) conducted a validation study of the STOP-Bang questionnaire for the identification of moderate/severe sleep apnea (AHI ≥ 15) in primary care in a Spanish population (Galicia-Spain). They recruited a sample of 178 people (57 women and 121 men) over 18 years old whose sociodemographic characteristics (age, gender, BMI) and selection criteria were similar to those of our study. They performed the diagnosis of OSA for the comparison of the STOP-Bang questionnaire score by using PSG and obtained for women a sensitivity of 93.8% (95% CI: 69.80–99.80) and a specificity of 63.4% (95% CI: 46.90–77.90), with an AUC of 0.816 (95% CI: 0.709–0.922) and an optimum cutoff point of 4, and for men a sensitivity of 55.2% (95% CI: 41.50–68.30) and a specificity of 73% (95% CI: 60.30–83.40), with an AUC of 0.686 (95% CI: 0.594–0.778) and an optimum cutoff of 6. Compared with the present study, the study conducted by Cruces-Artero et al. (15) showed better results in women both in sensitivity and specificity; however, in men, the sensitivity obtained by our study was higher and the specificity lower. This fact could be explained because Cruces-Artero et al. (15) used PSG to establish the diagnosis of sleep apnea and not HRP, developing their study for an AHI ≥ 15. In contrast, we used HRP to establish the diagnosis of sleep apnea in our study because it was performed entirely in the primary care setting, developing the study for an AHI > 15.

Saldias Penafiel et al. (21) recruited a sample of 205 Spanish-speaking people (95 men and 110 women) from a metropolitan area of Chile, with a mean age of 47.8 ± 20 years, who presented with clinical symptoms of sleep-related breathing disorders (habitual snoring and/or observed episodes of breathing pauses). The diagnosis of OSA was performed by using HRP. The STOP-Bang questionnaire achieved a sensitivity of 89% and a specificity of 36%, with an AUC of 0.67 for detecting moderate/severe apnea (AHI > 15). These results are consistent with those obtained in our study.

Similar results were also reported in the validation studies of the STOP-Bang questionnaire in the field of primary care in the non-Spanish language (22–26), with a mean sensitivity of 80.11% (range: 77.3–83.9) and a mean specificity of 61.46% (range: 52.8–66.35%).

Comparing the screening ability for moderate/severe apnea of the Berlin questionnaire, a widely used questionnaire whose sensitivity is 76% and its specificity is 59%, we observed that the STOP-Bang questionnaire (27) has a better ability to detect true positives.

## Conclusions

The STOP-Bang questionnaire, which consists of dichotomous questions, that is, its questions have only two possible answer options (Yes or No), is very useful as a screening tool. It allows people to populate it in ~1–2 min. It would be an easier tool than the Berlin questionnaire because the questions in the Berlin questionnaire are more difficult, given that they present polyatomic answers from 5 categories. The STOP-Bang questionnaire also shows acceptable internal consistency and good reproducibility.

The demonstrated good psychometric properties, and the ease of use make the STOP-Bang questionnaire an effective tool for screening moderate/severe OSA. Its use in primary care centers could contribute decisively to reducing the underdiagnosis that this disease presents today (3, 28), with the consequent impact on the complications inherent in the lack of treatment.

This study and the existing ones and the SEPAR recommendations that indicate the need to address OSA in primary care suggest that it is necessary to sensitize the primary care professionals to the use of measuring tools for the management of OSA. The STOP-Bang questionnaire is a recommendable tool for screening and diagnostic support.

Although the results support the existing evidence, we believe that it would be advisable to conduct new studies demonstrating the usefulness of the STOP-Bang questionnaire in different population groups in the primary care setting. This fact becomes even more necessary in the Spanish language version.

The development and validation of rapid and inexpensive tools for screening for sleep-related breathing disorders could facilitate their detection under the limited availability of time per patient of the primary health care professional.

## Weaknesses and strengths

Although we would have preferred to obtain a larger sample size, the situation produced by the coronavirus disease (COVID-19) pandemic significantly hampered the process of recruiting the study people, having to limit the fieldwork. Consequently, the precision of the parameters analyzed has been lower than desired, although above the estimated values in the sample size calculation.

Selection bias may have occurred because of the non-use of probabilistic sampling techniques. However, convenience or opportunistic sampling is commonly used in this type of validation study. As some authors have pointed out (29), it is necessary for validation studies that the selected sample covers a broad clinical spectrum of individuals, from the asymptomatic patient to the patient with symptoms specific to the condition being studied, to avoid overestimating the validity of the measuring tool. Given that the recruitment of patients was conducted in a population setting through the primary care services, we consider that this fact has been achieved, leading us to think that the possible selection bias was unimportant.

It is worth noting that, in our study, all the development of the study (recruitment of participants, completion of the STOP-Bang questionnaire, and completion of respiratory polygraphy) has been conducted in the primary care settings by medical and nursing health professionals.

Likewise, we understand the blinded process used as a strength of the study. We prevented the researcher who conducted the polygraphic analyzes from having prior knowledge of the results of the STOP-bang questionnaire, ensuring that the equality of the groups was maintained during the execution of the study. Thus, we reduced the risk of an information bias that could arise from the psychological influence of the knowledge of the interventions received in the groups among the study participants.

It is our intention, when the situation produced by the coronavirus disease (COVID-19) pandemic allows it, to increase the sample to obtain more precise results.

## Data availability statement

The original contributions presented in the study are included in the article/supplementary material, further inquiries can be directed to the corresponding author/s.

## Ethics statement

The studies involving human participants were reviewed and approved by Comité de ética de la investigación de Córdoba-España. The patients/participants provided their written informed consent to participate in this study.

## Author contributions

JS-M, LP-d-T, MV-A, RM-G, and EN-M contributed to conception and design of the study. JS-M organized the database and performed the statistical analysis. RM-G wrote the first draft of the manuscript. All authors contributed to manuscript revision, read, and approved the submitted version.

## Conflict of interest

The authors declare that the research was conducted in the absence of any commercial or financial relationships that could be construed as a potential conflict of interest.

## Publisher's note

All claims expressed in this article are solely those of the authors and do not necessarily represent those of their affiliated organizations, or those of the publisher, the editors and the reviewers. Any product that may be evaluated in this article, or claim that may be made by its manufacturer, is not guaranteed or endorsed by the publisher.
